# "My naturesound" - nature observations with sound recordings

**DOI:** 10.3897/BDJ.5.e20200

**Published:** 2017-10-20

**Authors:** Veljo Runnel, Marko Peterson, Allan Zirk

**Affiliations:** 1 University of Tartu Natural History Museum and Botanical Garden, Tartu, Estonia; 2 University of Tartu, Tartu, Estonia

**Keywords:** occurrence data, citizen science, biodiversity, sound recording, audio, multimedia, mobile application, Aves, Orthoptera, Amphibia

## Abstract

**Background:**

Online systems for observation reporting by citizen scientists have been operating for many years. iNaturalist (California Academy of Sciences 2016), eBird (Cornell Lab of Ornithology 2016) and Observado (Observation International 2016) are well-known international systems, Artportalen (Swedish Species Information Centre 2016) and Artsobservasjoner (Norwegian Biodiversity Information Centre 2016) are Scandinavian. In addition, databases and online solutions exist that are more directly research-oriented but still offer participation by citizen scientists, such as the PlutoF (University of Tartu Natural History Museum 2016) platform. The University of Tartu Natural History Museum maintains the PlutoF platform (Abarenkov et al. 2010) for storing and managing biodiversity data, including taxon observations. In 2014, development was started to integrate an observation app "Minu loodusheli"/"My naturesound" (University of Tartu Natural History Museum 2017b) (My naturesound, Fig. 1) within PlutoF system. In 2017, an English language version of the app (University of Tartu Natural History Museum 2017c) was launched that includes nearly all major sound-producing taxon groups in its taxonomy. The application also acts as a practical tool for collecting and publishing occurrence data for the Global Biodiversity Information Facility (Global Biodiversity Information Facility 2017) in standardized Darwin Core format together with download links to the multimedia files. Although the sound recording ability of mobile phones opens new opportunities to validate taxon occurrences, current technological solutions limit the use of recordings in biodiversity research.

The "My naturesound" allows the user to record taxon occurrences and to provide audio recordings as evidence. After installing the application, the user is promted to login with PlutoF system credentials or to register with PlutoF. The application is targeted primarely to citizen scientists, but researchers themselves can also use it as a tool for easy annotation of taxon occurrences.

**New information:**

The dataset consists observation data of birds, amphibians and insects by citizen scientists with on site audio recordings. The dataset gives the possibility to analyze the suitablility of mobile devices for recording animal vocalizations and their use in reporting.

## Introduction

The availability and affordability of smartphones have encouraged the use of mobile applications for nature observations. There are many well-known mobile applications that allow observers to annotate taxon occurrences and to add important data, such as date, time and coordinates, automatically from the mobile device itself, e.g. iNaturalist, eBird and Anymals+Plants (SiSol Systems 2015). As all modern smartphones are equipped with an easy to operate camera, adding a photo to a taxon observation is easy with plants, some mammals or less mobile insects. Photos can be used to aid identification, although the observer may not be aware of which aspect of the taxon is most important and needs to be clearly in focus or properly displayed. Taking mobile photos of distant animals provides much poorer resolution, thereby hindering indentification. While photography is widely used for observation applications (iNaturalist, eBird), audio recordings of nature observations are far less common. Although applications for mobile phones exist for use as the voice recorder (Samsung Voice Recorder, TapeMachine, Field Recorder etc on Android devices), few apps are used to provide an audio recording to a taxon observation. The benefits of this feature are obvious - even a mediocre sound recording of a bird hidden in the bushes can aid identification more than relying solely on a mobile photo at distance.

Although audio recording by mobile phones opens new opportunities for validation of biodiversity observations, there remain important limitations. Mobile phone sound processing is usually designed for human speech, thereby filtering certain frequencies. Also, recording capability is limited by the frequency response of the microphone unit and the sampling rate of the smartphone software (Brown and Evans 2011). We did not address these issues specifically in our project, but those using audio files recorded with mobile phones for research should be aware of these limitations.

"Minu loodusheli"/"My naturesound" is a mobile app developed by University of Tartu Natural History Museum in cooperation with the Estonian Natural History Museum. This app helps to annotate observations of a specific taxon and add a audio recording from the location of the observed specimen. The app is backed by the cloud database and workbench PlutoF, specifically designed for working with taxon occurrences and related biodiversity data. Both the mobile app and PlutoF workbench are also considered optimal for the European Biodiversity Observation Network (EU BON 2016) project. Collected data are published on the GBIF portal for download as Darwin Core Archive package, EML package or annotated GBIF data package.

The app is currently available for both Android and iOS platforms, but as the iOS version will not be updated for financial reasons, newer phones and operation system versions may not be compatible with the app.

## Project description

### Title

"My naturesound" - nature observations with sound recordings.

### Personnel

Observation data are collected by citizen scientists and other users of the application, observations are reviewed by professional biologists or volunteer naturalists recruited by University of Tartu Natural History Museum. As of August 2017, 186 individuals have contributed to the dataset. The full list of contributors and their individual input can be found on the project webpage (University of Tartu Natural History Museum 2017a).

### Study area description

Observation area is not restricted, but as most users live in Estonia, the observations are mostly from Estonia.

### Design description

The mobile application enables the user to store observation data: date (aquired automatically), location (aquired automatically if GPS is switched on, otherwise manually using map application) and taxon (species-level identification or higher taxon level identification). As the application is intended mostly for citizen scientists, there are no obligatory data fields for environmental parameters, although the user can add additional information as remarks. Moreover, the PlutoF workbench allows later addition of data fields when working with uploaded observation data. Recording and adding an audio file to the observation is a prerequisite for app data flow; adding an image is optional. All observation data are linked to the user's profile and are transmitted via data upload to the cloud database. The app can be used offline for recording therefore the availability of mobile networks will not limit the use of the app in the field. The app uses PlutoF customized API protocol to upload data (University of Tartu Natural History Museum 2016).

**Figure 2.**
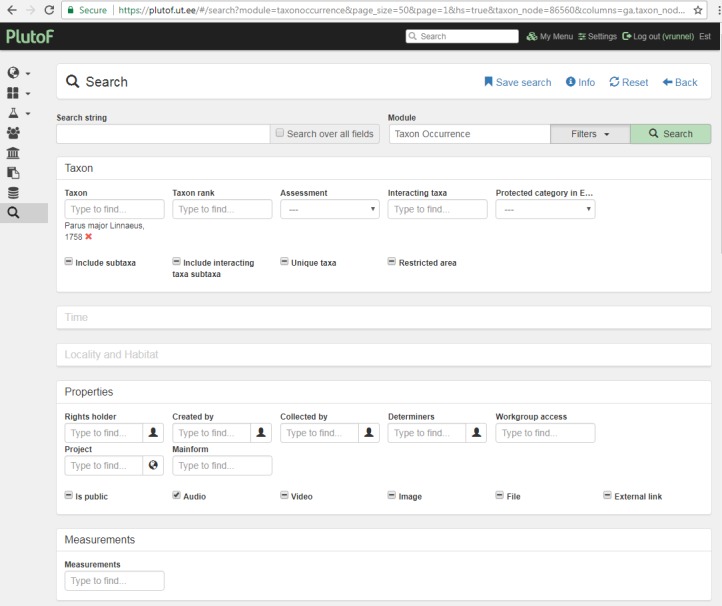
The search window of PlutoF workbench for Parus major observations with the audio files attached.

**Figure 3.**
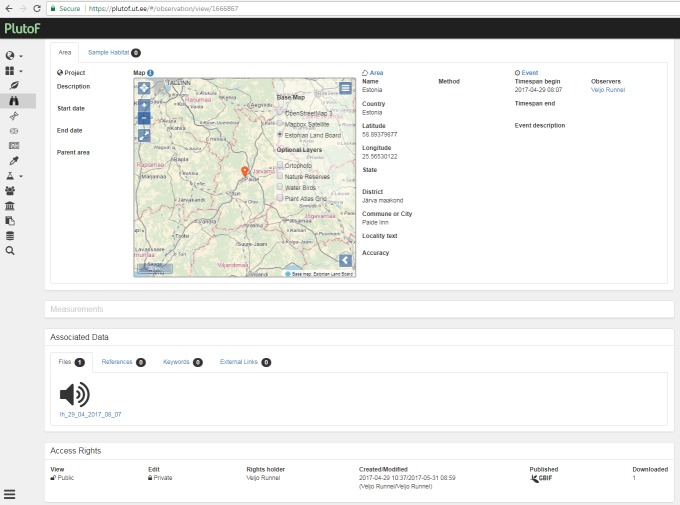
The menu item of the PlutoF workbench which shows how the audio file is linked to an observation record.

**Figure 4.**
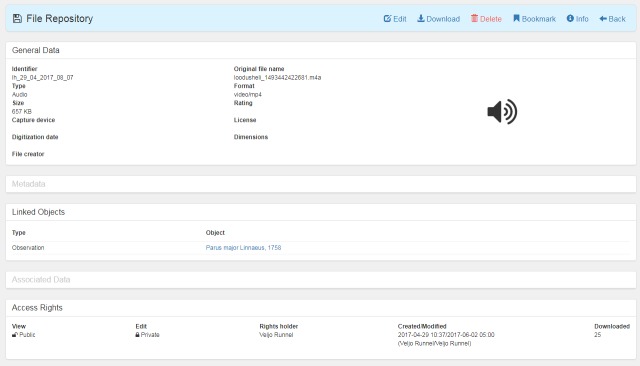
The properties of a multimedia file viewed on PlutoF workbench.

**Figure 5.**
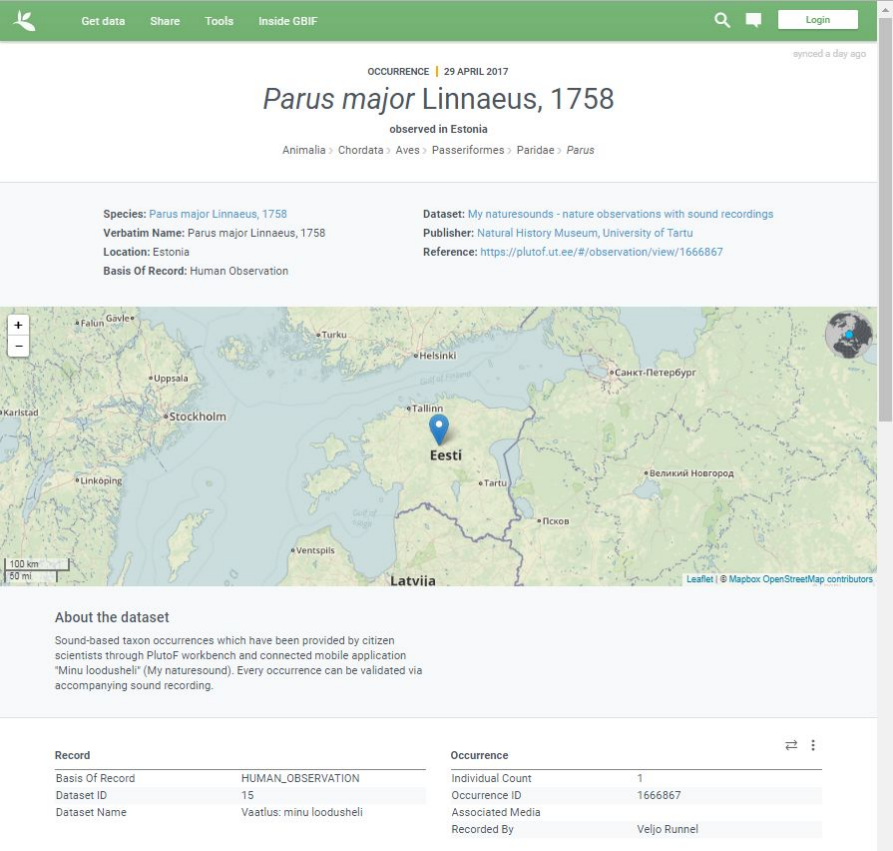
The details of an occurrence record viewed on GBIF platform.

**Figure 6.**
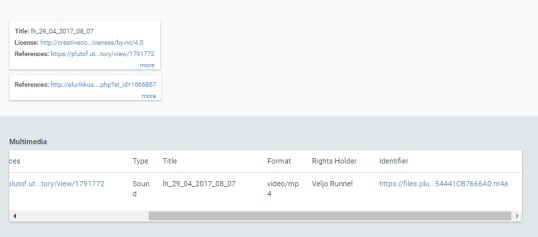
The details of a multimedia file viewed on GBIF platform.

Once the data have been stored in PlutoF cloud database and made publicly available, all registered database users can access the observation data and audio files for research purposes. For example, if a user executes a search for observations of Great Tit (*Parus major* L.) with audio files attached, all such observations obtained via "My naturesound" app will be also retrieved and displayed (Fig. 2). If a single observation record is opened, the audio file can be downloaded (Fig. 3) and analyzed in the user’s computer with appropriate software. The audio file properties - type, format, original name, size etc - can also be viewed on the PlutoF workbench when the file properties menu is open (Fig. 4). As retrieval of audio file metadata from mobile phones is less straightforward than image files, the metadata of audio file is limited. In addition, privacy issues concerning citizen science projects must be adhered to when retrieving information from users’ phones. As PlutoF biodiversity data are regularily published on GBIF, all aforementioned information can be also retrieved from the GBIF data portal (Fig. 5). Occurrence record view also includes multimedia file information with a link to the actual audio file, which can be listened to and downloaded (Fig. 6).

## Sampling methods

### Study extent

World

### Sampling description

Mostly observations by citizen scientists using mobile application to record coordinates and time of observation and adding audio recordings to the observation. Adding an audio file is obligatory when using the application, otherwise observations cannot be sent to database.

### Quality control

All observations include a audio recording used for species identification. Recordings are reviewed on the PlutoF workbench. Incorrect identifications are corrected; the observation with inappropriate recordings are rejected.

### Step description

Observations are recorded with the mobile app "Minu loodusheli" (My naturesound), observation records with audio files are transferred to the PlutoF cloud database. Observations are reviewed within PlutoF system by appointed persons.

## Geographic coverage

### Description

**Figure 7.**
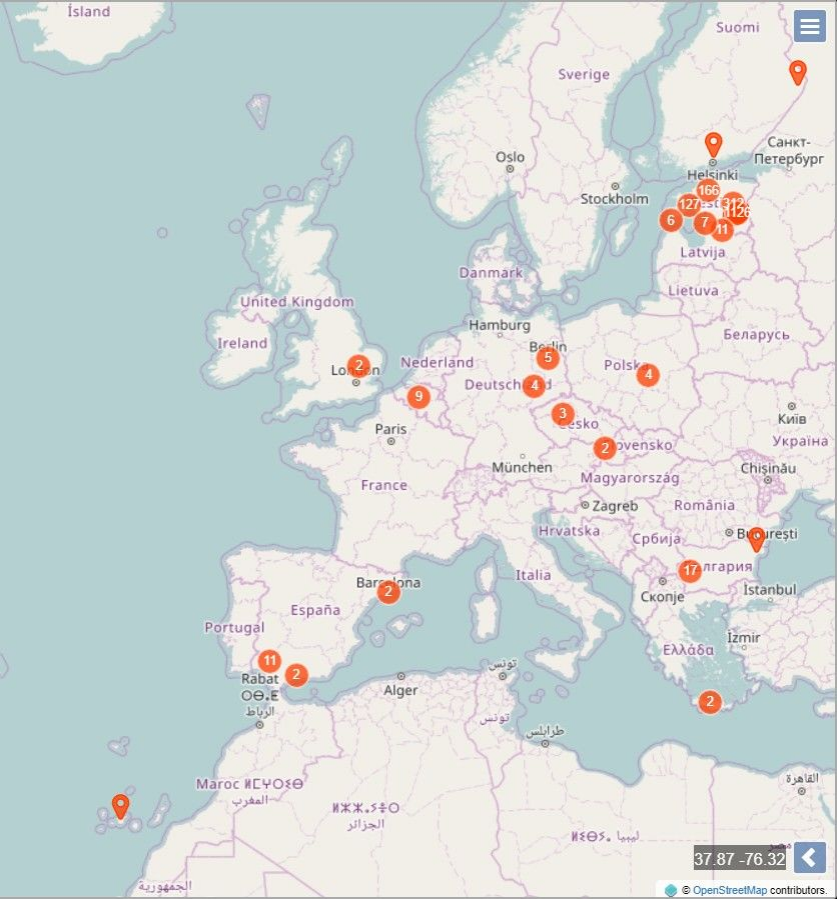
The occurrence map of "My naturesound" observations (as of August 2017).

App users can make observations throughout the world; there are no geographical limitations. As the promotion of the application is mostly in Estonian, majority of observations are also from Estonia (Fig. 7).

## Taxonomic coverage

### Taxa included

**Table:** 

Rank	Scientific Name	
kingdom	Aves	
kingdom	Anura	
kingdom	Insecta	
kingdom	Mammalia	

## Temporal coverage

### Notes

2015-02-21 through 2017-09-25

## Usage rights

### Use license

Creative Commons Public Domain Waiver (CC-Zero)

### IP rights notes

Creative Commons Attribution Non Commercial (CC-BY-NC) 4.0 License

## Data resources

### Data package title

"My naturesound" - nature observations with sound recordings

### Resource link

http://doi.org/10.15156/bio/587439

### Alternative identifiers

cb6e66f1-3056-404d-a341-bb856762c57c

### Number of data sets

2

### Data set 1.

#### Data set name

"My naturesound" - nature observations with sound recordings

#### Data format

Darwin Core

#### Number of columns

59

#### Download URL

https://plutof.ut.ee/ipt/archive.do?r=naturesound

#### Data format version

1.9

#### Description

Sound-based taxon occurrences provided by citizen scientists through the PlutoF workbench and connected mobile application "Minu loodusheli" (My naturesound). Every occurrence can be validated via accompanying audio recording. The dataset consists of two tables which for practical reasons are considered as individual datasets - one for occurrences (59 data columns) and the other for audio files linked to each occurrence (12 data columns). Multimedia files are referred in the data table by url and can be downloaded individually through the PlutoF workbench if the file owner has granted access to the files. All information is in English, except the "occurrenceRemarks" and "identificationRemarks" columns, which can be in set to the application user’s choice.

**Data set 1.** 

Column label	Column description
id	Record ID
type	The nature or genre of the resource. For Darwin Core, recommended best practice is to use the name of the class that defines the root of the record.
modified	The most recent date-time on which the resource was changed.
language	A language of the resource.
license	A legal document giving official permission to do something with the resource.
rightsHolder	A person or organization owning or managing rights over the resource.
references	A related resource that is referenced, cited, or otherwise pointed to by the described resource.
datasetID	An identifier for the set of data.
datasetName	The name identifying the data set from which the record was derived.
basisOfRecord	The specific nature of the data record (for current dataset - HumanObservation)
informationWithheld	Additional information that exists, but that has not been shared in the given record.
occurrenceID	An identifier for the Occurrence (as opposed to a particular digital record of the occurrence).
occurrenceRemarks	Comments or notes about the Occurrence.
recordedBy	A list (concatenated and separated) of names of people, groups, or organizations responsible for recording the original Occurrence.
individualCount	The number of individuals represented present at the time of the Occurrence.
sex	The sex of the biological individual(s) represented in the Occurrence.
behavior	A description of the behavior shown by the subject at the time the Occurrence was recorded.
associatedMedia	A list (concatenated and separated) of identifiers (publication, global unique identifier, URI) of media associated with the Occurrence.
associatedReferences	A list (concatenated and separated) of identifiers (publication, bibliographic reference, global unique identifier, URI) of literature associated with the Occurrence.
associatedTaxa	Secondary taxa, associated with current occurrence record
eventID	An identifier for the set of information associated with an Event (something that occurs at a place and time).
eventDate	The date-time or interval during which an Event occurred.
verbatimEventDate	The verbatim original representation of the date and time information for an Event.
habitat	A category or description of the habitat in which the Event occurred.
locationID	An identifier for the set of location information (data associated with dcterms:Location).
country	The name of the country or major administrative unit in which the Location occurs.
countryCode	The standard code for the country in which the Location occurs. Recommended best practice is to use ISO 3166-1-alpha-2 country codes.
stateProvince	The name of the next smaller administrative region than country (state, province, canton, department, region, etc.) in which the Location occurs.
county	The full, unabbreviated name of the next smaller administrative region than stateProvince (county, shire, department, etc.) in which the Location occurs.
municipality	The full, unabbreviated name of the next smaller administrative region than county (city, municipality, etc.) in which the Location occurs. Do not use this term for a nearby named place that does not contain the actual location.
locality	The specific description of the place. Less specific geographic information can be provided in other geographic terms (higherGeography, continent, country, stateProvince, county, municipality, waterBody, island, islandGroup). This term may contain information modified from the original to correct perceived errors or standardize the description.
minimumElevationInMeters	The lower limit of the range of elevation (altitude, usually above sea level), in meters.
maximumElevationInMeters	The upper limit of the range of elevation (altitude, usually above sea level), in meters.
minimumDepthInMeters	The lesser depth of a range of depth below the local surface, in meters.
maximumDepthInMeters	The greater depth of a range of depth below the local surface, in meters.
decimalLatitude	The geographic latitude (in decimal degrees, using the spatial reference system given in geodeticDatum) of the geographic center of a Location. Positive values are north of the Equator, negative values are south of it. Legal values lie between -90 and 90, inclusive.
decimalLongitude	The geographic longitude (in decimal degrees, using the spatial reference system given in geodeticDatum) of the geographic center of a Location. Positive values are east of the Greenwich Meridian, negative values are west of it. Legal values lie between -180 and 180, inclusive.
geodeticDatum	The ellipsoid, geodetic datum, or spatial reference system (SRS) upon which the geographic coordinates given in decimalLatitude and decimalLongitude as based. Recommended best practice is use the EPSG code as a controlled vocabulary to provide an SRS, if known. Otherwise use a controlled vocabulary for the name or code of the geodetic datum, if known. Otherwise use a controlled vocabulary for the name or code of the ellipsoid, if known. If none of these is known, use the value "unknown".
coordinateUncertaintyInMeters	The horizontal distance (in meters) from the given decimalLatitude and decimalLongitude describing the smallest circle containing the whole of the Location. Leave the value empty if the uncertainty is unknown, cannot be estimated, or is not applicable (because there are no coordinates). Zero is not a valid value for this term.
identificationID	An identifier for the Identification (the body of information associated with the assignment of a scientific name). May be a global unique identifier or an identifier specific to the data set.
identifiedBy	A list (concatenated and separated) of names of people, groups, or organizations who assigned the Taxon to the subject.
dateIdentified	The date on which the subject was identified as representing the Taxon. Recommended best practice is to use an encoding scheme, such as ISO 8601:2004(E).
identificationRemarks	Comments or notes about the Identification. Can be in users' native language.
taxonID	An identifier for the set of taxon information (data associated with the Taxon class). May be a global unique identifier or an identifier specific to the data set.
scientificNameID	An identifier for the nomenclatural (not taxonomic) details of a scientific name.
scientificName	The full scientific name, with authorship and date information if known. When forming part of an Identification, this should be the name in lowest level taxonomic rank that can be determined. This term should not contain identification qualifications, which should instead be supplied in the identificationQualifier term.
higherClassification	A list (concatenated and separated) of taxa names terminating at the rank immediately superior to the taxon referenced in the taxon record.
kingdom	The full scientific name of the kingdom in which the taxon is classified.
phylum	The full scientific name of the phylum or division in which the taxon is classified.
class	The full scientific name of the class in which the taxon is classified.
order	The full scientific name of the order in which the taxon is classified.
family	The full scientific name of the family in which the taxon is classified.
genus	The full scientific name of the genus in which the taxon is classified.
subgenus	The full scientific name of the subgenus in which the taxon is classified. Values should include the genus to avoid homonym confusion.
specificEpithet	The name of the first or species epithet of the scientificName.
infraspecificEpithet	The name of the lowest or terminal infraspecific epithet of the scientificName, excluding any rank designation.
taxonRank	The taxonomic rank of the most specific name in the scientificName. Recommended best practice is to use a controlled vocabulary.
verbatimTaxonRank	The taxonomic rank of the most specific name in the scientificName as it appears in the original record.
vernacularName	A common or vernacular name.

### Data set 2.

#### Data set name

Multimedia files linked to the "My naturesound" observations

#### Number of columns

12

#### Download URL

https://plutof.ut.ee/ipt/archive.do?r=naturesound

#### Description

The data table describing the properties of audio files which are linked to the "My naturesound" observations.

**Data set 2.** 

Column label	Column description
id	Identifier of the resource
type	The nature or genre of the resource.
format	The file format, physical medium, or dimensions of the resource.
identifier	An unambiguous reference to the resource within a given context.
references	A related resource that is referenced, cited, or otherwise pointed to by the described resource.
title	A name given to the resource.
description	An account of the resource.
created	Date of creation of the resource.
creator	An entity primarily responsible for making the resource.
source	A related resource from which the described resource is derived.
license	A legal document giving official permission to do something with the resource.
rightsHolder	A person or organization owning or managing rights over the resource.

## Additional information

Natural History Museum, University of Tartu: "My naturesound" - nature observations with sound recordings.

## Figures and Tables

**Figure 1.**
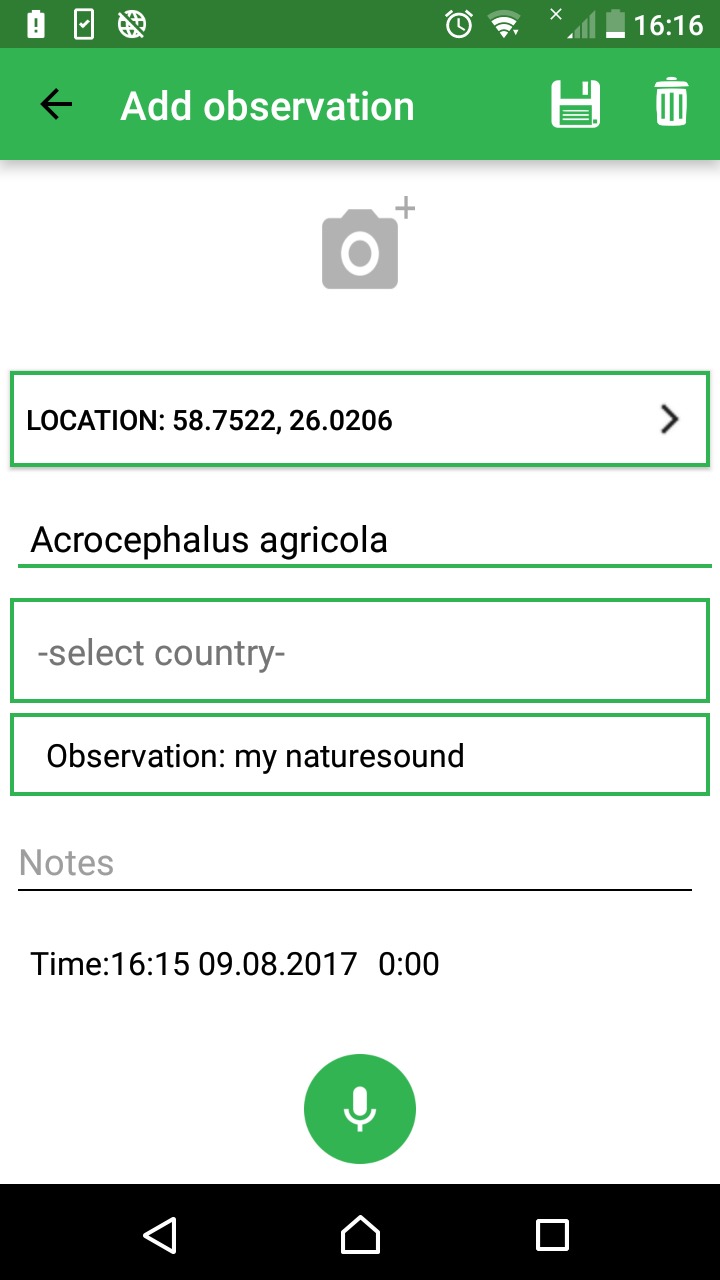
The application interface on the Android patform.
